# First complete genome sequence of *Tenacibaculum maritimum* serotype O4, a rising threat in marine aquaculture

**DOI:** 10.1128/mra.01122-24

**Published:** 2024-12-27

**Authors:** M. Pilar Escribano, Rubén Salvador-Clavell, Miguel Balado, Beatriz Magariños, Carmen Amaro, Manuel L. Lemos

**Affiliations:** 1 Department of Microbiology and Parasitology, Aquatic One Health Research Center (ARCUS) and Faculty of Biology-CIBUS, University of Santiago de Compostela 16780 , Santiago de Compostela, A Coruña, Spain; 2 Department of Microbiology and Ecology and BIOTECMED Institute, University of Valencia, Valencia, Spain; Montana State University, Bozeman, Montana, USA

**Keywords:** *Tenacibaculum maritimum*, tenacibaculosis, fish pathogens

## Abstract

*Tenacibaculum maritimum* is a Gram-negative marine fish pathogen. Here, we present the complete genome sequence of strain SP9.1, representing the emerging serotype O4. Selected as a model for pathogenesis studies, this genome provides valuable insights into the genetic basis of virulence and adaptation, complementing existing data for serotype O1.

## ANNOUNCEMENT

*Tenacibaculum maritimum* (family *Flavobacteriaceae*, class *Flavobacteriia*, and phylum *Bacteroidota*) is the causative agent of marine tenacibaculosis, a disease that significantly impacts fish aquaculture facilities worldwide (1). The type IX secretion system (2), adhesion mechanisms (3), tissue-degrading enzymes, outer membrane vesicles (OMVs) (4), and iron uptake mechanisms (5) have been proposed as contributors to the virulence of this pathogen. However, the specific molecular factors involved in pathogenesis remain unidentiﬁed and could be further explored using omics approaches. Within *T. maritimum*, four serotypes and eight subtypes have been documented (6), although the only complete genome sequence published to date corresponds to a serotype O1 strain (2). Notably, an increasing prevalence of serotype O4 strains has been reported (7). To address this, the genome of *T. maritimum* SP9.1, representing serotype O4, has been sequenced.

Strain SP9.1 was previously isolated from diseased Atlantic salmon (*Salmo salar*) in Northwestern Spain and preserved at −80°C in cryovials containing *Flexibacter maritimus* medium (FMM; Condalab, Spain) supplemented with 8% glycerol (8). For sequencing, a single colony from an FMM agar plate, incubated at 28°C for 24 h, was used to inoculate 5 mL of FMM broth, which was also incubated at 28°C and 180 rpm for 24 h. Genomic DNA was extracted using the MagAttract HMW DNA kit (Qiagen, Germany), following the manufacturer’s protocol for “Purification of High-Molecular-Weight Genomic DNA from Gram-Negative Bacteria.” The DNA integrity was verified by electrophoresis and Nanodrop analysis, and its concentration was quantified using an Invitrogen Qubit 3.0 fluorometer (Thermo Fisher Scientific, USA). Library preparation and sequencing were performed on a PacBio Sequel IIe platform (10 kb library) by the Central Support Service for Experimental Research (SCSIE) at the University of Valencia (Valencia, Spain). The SMRTbell Express Template Preparation Kit v3.0 was used for library construction. Briefly, DNA shearing was performed by centrifugation using Covaris g-Tubes, producing fragments of 7–10 kb. Then, a cleanup with 1× SMRT bell cleanup beads was done. The PacBio sequencing was performed following the instructions of Sequel II sequencing kit 2.0 and SMRT cell 8M tray. The PacBio sequencing yielded 343,665 reads with a Q20 score. A *de novo* genome assembly was performed using Flye version 2.9-b1768 with a long-read-only assembly approach (9) using the Hi-Fi reads from PacBio.

**Fig 1:**
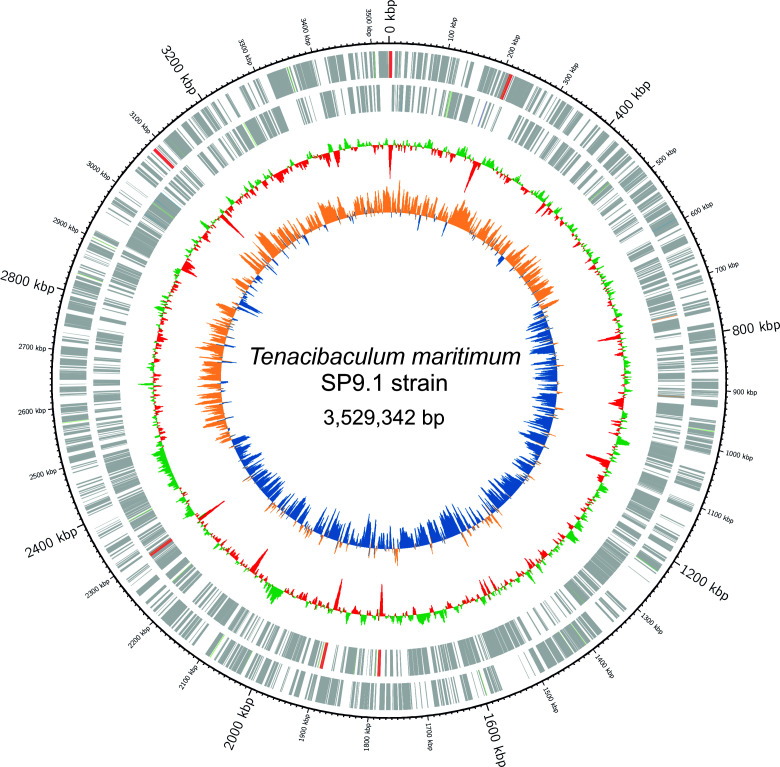
Circular representation of the SP9.1 genome. Protein-coding genes (CDS) and rRNA are shown on the two outer rings representing the forward and reverse strands from outer to inner, colored gray or soft red, respectively. The innermost ring indicates the GC content per sliding window, with green indicating above-average and red indicating below-average content. The second ring shows GC skew, with orange and blue representing the skew.

The draft genome consisted of a single contig with a total length of 3,529,342 bp and 3,195 genes, from which 3,084 are coding DNA sequences (CDSs) (Table 1) (Fig. 1). Analysis with Genomad confirmed the presence of a single chromosome and suggested that no plasmids or phages are present in the genome. The curated data set was annotated, and open reading frames were identified using Bakta v1.8.2 (10) and RAST (11), resulting in 3,084 protein-coding genes. The GC content of the contig was 31.97%. These *in silico* predictions provide insights into the functional role of key components in serotype O4 strains and will serve as a valuable reference for future whole-genome-based molecular epidemiology studies focused on tenacibaculosis emergence and spread in marine fish.

**TABLE 1:** Genome statistics for *Tenacibaculum maritimum* SP9.1 isolate from Spain

Parameter	*Tenacibaculum maritimum* SP9.1
Sample source	Atlantic salmon (*Salmo salar*)
Place of isolation	Northwestern Spain
Total genome size (bp)	3,529,342
Number of contigs	1
*N*_50_ (bp)	3,529,342
Genome coverage (×)	554
GC content (%)	31.97
Genes (Total)	3,195
Coding DNA sequences	3,084
Complete rRNAs	6, 6, 6 (5S, 16S, 23S)
tRNA	57
Accession No.	CP170088
BioProject	PRJNA1159549
BioSample	SAMN43577918

## Data Availability

This Whole-Genome Shotgun project has been deposited in DDBJ/ENA/GenBank under accession no. SAMN43577918 under NCBI BioProject PRJNA1159549 (accession number CP170088). The version described in this paper is the first version CP170088.1. The raw reads have been deposited in the SRA database with accession number SRX26369952.
